# The functions of SET domain bifurcated histone lysine methyltransferase 1 (SETDB1) in biological process and disease

**DOI:** 10.1186/s13072-023-00519-1

**Published:** 2023-12-07

**Authors:** Hanshen Luo, Xingliang Wu, Xue-Hai Zhu, Xin Yi, Dunfeng Du, Ding-Sheng Jiang

**Affiliations:** 1 Division of Cardiovascular Surgery, Tongji Hospital, Tongji Medical College, Huazhong University of Science and Technology, 1095 Jiefang Ave., Wuhan, 430030 China; 2 Department of Cardiology, Renmin Hospital of Wuhan University, Wuhan, China; 3 Key Laboratory of Organ Transplantation, Ministry of Education; NHC Key Laboratory of Organ Transplantation; Key Laboratory of Organ Transplantation, Chinese Academy of Medical Sciences, Wuhan, Hubei China; 4 Institute of Organ Transplantation, Tongji Hospital, Tongji Medical College, Huazhong University of Science and Technology, Wuhan, Hubei China

**Keywords:** SETDB1, Histone methylation, Non-histone proteins methylation, Protein methylation, Heterochromatin, Cancer

## Abstract

Histone methyltransferase SETDB1 (SET domain bifurcated histone lysine methyltransferase 1, also known as ESET or KMT1E) is known to be involved in the deposition of the di- and tri-methyl marks on H3K9 (H3K9me2 and H3K9me3), which are associated with transcription repression. SETDB1 exerts an essential role in the silencing of endogenous retroviruses (ERVs) in embryonic stem cells (mESCs) by tri-methylating H3K9 (H3K9me3) and interacting with DNA methyltransferases (DNMTs). Additionally, SETDB1 is engaged in regulating multiple biological processes and diseases, such as ageing, tumors, and inflammatory bowel disease (IBD), by methylating both histones and non-histone proteins. In this review, we provide an overview of the complex biology of SETDB1, review the upstream regulatory mechanisms of SETDB1 and its partners, discuss the functions and molecular mechanisms of SETDB1 in cell fate determination and stem cell, as well as in tumors and other diseases. Finally, we discuss the current challenges and prospects of targeting SETDB1 for the treatment of different diseases, and we also suggest some future research directions in the field of SETDB1 research.

## Introduction

Protein methylation, a covalent modification on proteins, is dynamically regulated by protein methyltransferases and demethylases, and *S*-adenosyl-l-methionine (AdoMet) is the main methyl group donor [1–3]. Protein methylation occurs mainly at lysine or arginine residues of histones and non-histones and has been demonstrated to be implicated in the regulation of many biological processes by affecting the activity, subcellular localization, or stability of proteins [4–7]. In recent years, major advancements in our understanding of protein methylation have been made, including not only its regulatory mechanisms, but also its pathophysiological functions [8–11].

The SET domain bifurcated histone lysine methyltransferase 1 (SETDB1), also known as lysine *N*-methyltransferase 1E (KMT1E) or Erg-associated SET domain (ESET), is a family member of the SET domain-containing histone methyltransferases. SETDB1 deposits di- and tri-methyl marks on H3K9 (H3K9me2 and H3K9me3) which are transcriptional repression marks [12–16]. Additionally, SETDB1 has been found to methylate non-histones, such as tri-methylating AKT at K64 and K140 (AKT^K64me3^ and AKT^K140me3^), and di-methylating P53 at K370 (P53^K370me2^) [17–19]. SETDB1 has been shown to be involved in maintaining endogenous retroviruses (ERVs) silencing in embryonic stem cells (mESCs) [20–23], as well as in cell fate determination and tumorigenesis [24–29]. In this review, we summarize the structure features of SETDB1, the upstream regulatory mechanisms controlling SETDB1 expression and activity, and the partners of SETDB1, as well as the pivotal roles of SETDB1 in cancer progression, inflammatory bowel disease, ageing, and embryonic stem cells by regulating methylation of H3K9 and non-histone proteins.

## Structure of SETDB1

**Fig. 1:**
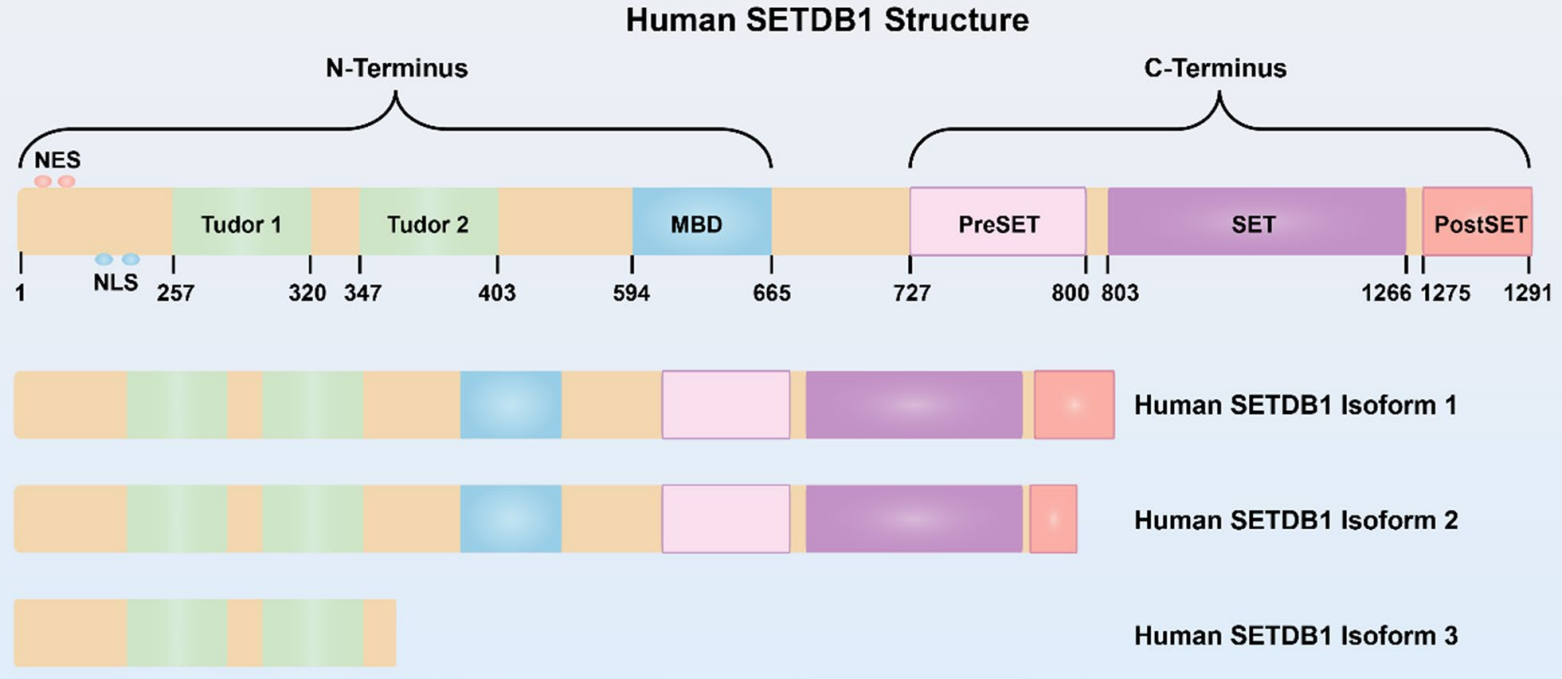
Schematic representation of human SETDB1 and its isoforms. The domains are indicated as different colors, and the SET domain is the major catalytic domain. *NES* nuclear export sequence, *NLS* nuclear localization sequence, *MBD* methyl-CpG binding domain, *SET* Su(var)3–9, Enhancer-of-zeste and Trithorax

SETDB1 was first identified by Harte et al*.* in 1999, and they revealed that *Setdb1* gene localized to human chromosome band 1q21 [30]. By using the N-terminal region of ERG as a bait to screen a yeast two-hybrid mouse cDNA library, Yang et al*.* isolated a 4.6 kb full-length mouse cDNA encoding a protein of 1307 amino acids, referred to as ESET [31]. Mouse ESET has 92% similarity to human protein SETDB1, and SETDB1 is a histone H3K9-specific methyltransferase contributing to heterochromatin protein 1a (HP1a)-mediated silencing of euchromatic genes [16, 32]. The evolutionarily conserved SET, pre-SET, and post-SET domains comprise the C-terminal region of SETDB1, which is necessary for its methyltransferase activity. The N-terminal region of SETDB1 contains two consecutive tudor domains (TUDs) and a methyl-CpG binding domain (MBD) that interacts with chromatin modifying enzymes, such as DNA methyltransferases (DNMTs), to participate in DNA silencing [12, 33, 34] (Fig. 1). Three isoforms of *Setdb1* gene have been identified, of which isoform 1 is encoded by the longest transcript containing all intact domains, and is widely expressed [32, 35]. Although isoform 2 is a shorter splice variant, it still has all the important domains similar to those of isoform 1. However, in contrast to isoform 1, isoform 3 contains only 400 amino acids at the N-terminus (Fig. 1) [12, 33].

## The upstream regulatory mechanisms of SETDB1

**Fig. 2:**
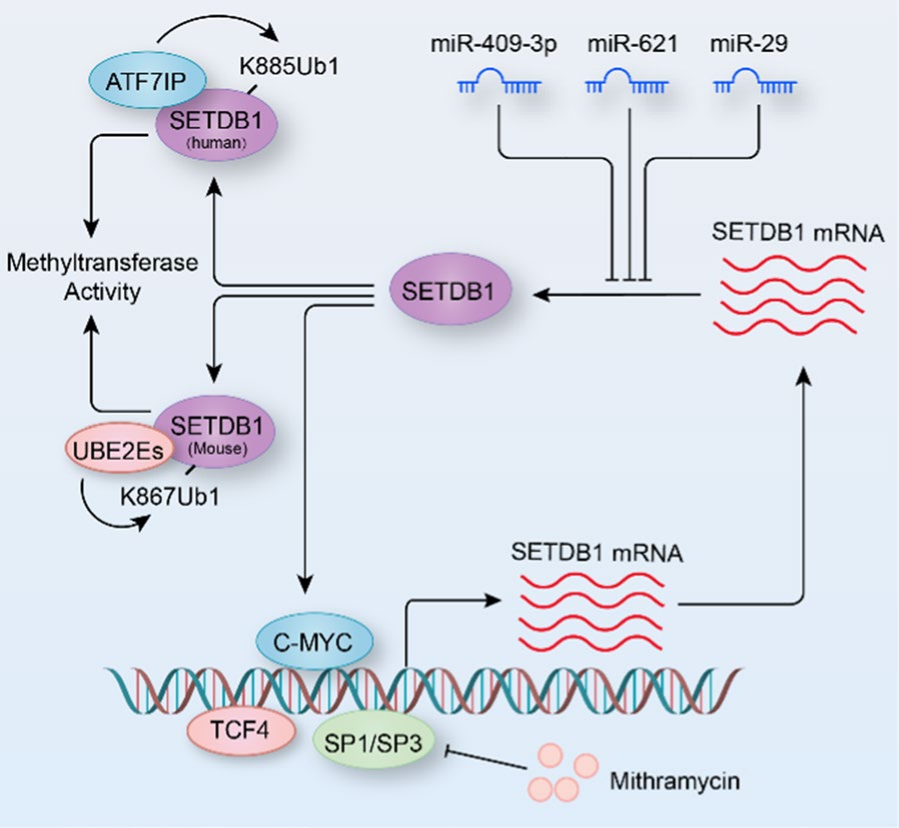
The upstream regulatory mechanisms of SETDB1. The expression of SETDB1 is regulated by transcription factors TCF4, C-MYC, and SP1/SP3. miR-409-3p, miR-621, and miR-29 directly target the 3′-UTR of Setdb1 and inhibits its expression. ATF7IP and UBE2Es directly interact with and monoubiquitinate SETDB1 to enhance the methyltransferase activity of SETDB1. Mithramycin is an inhibitor of SP1/SP3

The expression and activity of SETDB1 is regulated at multiple levels (Fig. 2). Compared with control, the protein levels of SETDB1 and its substrate H3K9me3 are obviously increased in the striatal neurons of Huntington’s disease patients and transgenic R6/2 (a Huntington’s disease mouse model) mice, and both specificity protein 1 (Sp1) and Sp3 can bind to the *Setdb1* promoter to activate the transcription of *Setdb1* gene [36]. Mithramycin is an antibiotic agent that has been revealed to suppress the growth of cancers by preventing the binding of Sp-family transcription factors to the DNA of gene promoters [37]. Unsurprisingly, mithramycin inhibits the basal promoter activity of *Setdb1* gene in a dose-dependent manner, and in addition, combined treatment with mithramycin and cystamine extends the lifespan of R6/2 mice by 40% and obviously improves the behavioral and neuropathological phenotype [36]. In addition, both mithramycin A and its analog (mithralog) EC-8042 effectively suppress SETDB1 expression in melanoma cells and enhance the efficacy of mitogen-activated protein kinase-inhibitor-based therapies for melanoma [38]. In gastric cancer, transcription factor 4 (TCF4) directly bind to the promoter of *Setdb1* gene (binding motif, CAAAG) to enhance the expression of SETDB1, and approximately 90% of patients with gastric cancer (GC) are caused by *Helicobacter pylori* infection, which promotes SETDB1 expression in a TCF4-dependent manner [39]. Furthermore, elevated SETDB1 interacts with ERG to promote gastric carcinogenesis and metastasis by binding to the promoter regions of matrix metalloproteinase 9 (MMP9) and cyclin D1 (CCND1) to accelerate their transcription [39]. In breast cancer cells, SETDB1 increases the expression of c-MYC to promote cell cycle progression, enhanced growth, and colony formation, and increased c-MYC positive feedback regulates the expression of SETDB1 by directly binding to *Setdb1* promoter and enhances its transcription [40].

MicroRNAs are a class of small non-coding RNAs that negatively regulate gene expression by targeting the 3′-UTRs of mRNAs and causing their mRNAs degradation [41]. Under certain conditions, microRNAs can affect the expression of SETDB1 by targeting its mRNA. For example, miR-621 and miR-29 bind directly to the 3′-UTR of *Setdb1* and inhibit its expression. Moreover, the miR-621-SETDB1 strengthens radiosensitivity in hepatocellular carcinoma (HCC) through activation of the p53 signaling pathway [42]. miR-409-3p has been reported to negatively regulate the expression of SETDB1 in non-small cell lung cancer (NSCLC) [43].

The methyltransferase activity, stability, and subcellular localization of SETDB1 protein are essential for its function. For example, SETDB1 in the nucleus is necessary for the expansion of adult muscle stem cells and the suppression of skeletal myoblast terminal differentiation, and Wnt3a-dependent cytoplasmic SETDB1 relocalization and genomic release from certain target genes promote myogenesis [44]. Activating transcription factor 7-interacting protein (ATF7IP) mediates nuclear retention of SETDB1 by binding to the N-terminal region of SETDB1 to inhibit its nuclear export, and increase the K885 ubiquitination of SETDB1 to enhance its methyltransferase activity in HEK293T cells [45]. The deficiency of ATF7IP facilitates proteasomal degradation of nuclear SETDB1 protein, implying that stability of SETDB1 regulated by ATF7IP is essential for heterochromatin formation [46]. Furthermore, the disruption of either SETDB1 or ATF7IP in tumor cells restores tumor antigen expression and augments tumor immunogenicity [47]. Similar to K885 ubiquitination of SETDB1 in humans, the K867 in the SET-insertion domain of SETDB1 can also be monoubiquitinated by UBE2E family of E2 enzymes in an E3-independent manner in mESCs, which is indispensable for methyltransferase activity of SETDB1 and its role in endogenous retrovirus silencing [48]. The monoubiquitinated Egg/Eggless (the ortholog of SETDB1 in *Drosophila*) is required for piRNA-mediated transposon repression [49]. Windei/Wed (ATF7IP ortholog in *Drosophila*) controls nuclear retention of Egg/Eggless and recruits mUb-Egg to transposon loci for silencing [49]. These studies demonstrate that the monoubiquitination of SETDB1 is critical for its methyltransferase activity, nuclear localization, and function. Therefore, further identification of the enzymes that regulate the monoubiquitination of SETDB1 and the partners that control its nuclear localization is vital for exploring the biological function of SETDB1.

## The partners of SETDB1

As is well known, H3K9 can be methylated by a variety of histone methyltransferases [50, 51], and how these methyltransferases work together to dynamically regulate the methylation of H3K9 is a topic worthy of further investigation. Recent studies have shown that certain H3K9 methyltransferases, such as SETDB1/KMT1E, G9a/KMT1C, Suv39h1/KMT1A, and GLP/KMT1D, cooperate to form a megacomplex and function in gene silencing [52]. Methylation of H3K9 is usually linked to heterochromatin formation and gene silencing, and H3K9me3 is highly enriched in heterochromatic regions, while H3K9me1/2 is enriched in silent euchromatin regions [52]. HP1a is a common partner of SETDB1 and is essential for the formation and maintenance of heterochromatin in both *Drosophila* and mammals [53–56]. In *Drosophila*, mutants with hypomorphic or null expression of dSETDB1 result in the loss of most H3K9 methylation marks and HP1-binding on chromosome 4, a condensed heterochromatin region [57]. SETDB1, HP1a, and Su(var)3–9 inhibit the same genes on chromosome 4, and genes that non-ubiquitously expressed are preferentially targeted, and then stimulate genes in pericentromeric regions [53]. Furthermore, Maksimov et al*.* revealed that in *Drosophila*, Su(var)3–9 binds to the majority of single-copy genes in euchromatin only in the presence of dSETDB1, but is largely dSETDB1-independent at repeated sequences in heterochromatin [58, 59]. Similar to *Drosophila*, SETDB1 physically associates with HP1 and KAP1 around the euchromatic promoter, establishing a silenced state that is epigenetically heritable in mammalian cells [54]. Moreover, HP1 interaction-defective Setdb1 protein is subject to protein degradation by the proteasome pathway in mESCs, and HP1 mutants unable to recognize H3K9me2/3 or dimerize fail to stabilize Setdb1 [56]. HP1 deficiency results in the loss of pluripotency of mESCs and a reciprocal gain of lineage-specific features, which can be restored by overexpression of Setdb1, Nanog and Oct4 [55]. In addition, the heterochromatic chromatin assembly factor 1 (CAF1)-HP1a-SETDB1 complex monomethylates K9 on non-nucleosomal histone H3, which may subsequently trimethylated by SUV39h1/h2 in pericentric regions in HeLa cell line [60], and KRAB-ZFP-associated protein 1 (KAP-1) is a molecular scaffold that coordinates histone methylation and deposition of HP1 protein to repress gene expression [16]. Heterochromatin-inducible activity is inhibited by mTOR-mediated phosphorylation on KAP1, and KAP1 knockdown or drug-induced phosphorylation of KAP1 can force human cytomegalovirus out of latency in human hematopoietic stem cell [61]. In addition, Müller et al*.* reported that SETDB1 recruits the chromodomain protein M-phase phosphoprotein 8 (MPP8) to its genomic target loci and maintains transcriptional repression of LINE1 elements without preserving H3K9me3 levels, which is critical for maintaining self-renewal of ground-state pluripotent stem cells [62].

H3K9 methylation is associated with DNA methylation, which is inherited after mitosis in a manner coupled to DNA methylation, suggesting that SETDB1 may function in conjunction with DNMTs [52]. Deletion of *Setdb1* reduces the levels of H3K9me3 and loci-specific DNA methylation, while increasing 5-hydroxymethylation (5hmC) and binding of ten-eleven translocation 1 in mESCs [63]. The silencing of hypermethylated germline “genome-defence” genes is dependent upon SETDB1, PRC1.6/RING1B and DNA methylation in epiblast-like cells [64]. In contrast, in pre-implantation embryos and naïve ESCs, H3K9me3 and RING1B-dependent H2A^K119ub1^ are enriched at the hypomethylated promoters of germline genes that bind by the PRC1.6 DNA-binding subunits MGA/MAX/E2F6 [64]. These studies demonstrated that SETDB1 usually functions in the euchromatic region with multiple partners, and represses gene expression. Interestingly, recent studies suggest that DNA methylation does not appear to contribute to the maintenance of H3K9me3, as *Dnmt1* KO cells that have greatly reduced levels of DNA methylation on endogenous retroviruses can still maintain H3K9me3 [65]. Long terminal repeat (LTR) expression correlated with loss of H3K9me3, but loss of H3K9me3 did not always lead to transcriptional activation, probably due to DNA methylation [66, 67]. Therefore, the relationship between SETDB1 (H3K9me3) and DNA methylation is still controversial and more studies are needed for in-depth investigation.

## The functions of SETDB1 in biological processes and diseases

Current studies have shown that H3K9 is the main substrate of SETDB1, and SETDB1 regulates multiple biological processes (e.g., embryonic stem cell and aging) and various diseases (e.g., tumors and inflammatory bowel disease) by tri-methylating H3K9.

### SETDB1 in cell fate determination and stem cell

#### Cell fate determination

Meiosis is a biological process in which diploid germ cells undergo one round of DNA replication followed by two rounds of division to produce haploid gametes [68]. Chen et al*.* demonstrated that SETDB1-catalyzed H3K9me3 is indispensable for the formation of bivalent in early meiosis [68]. Spermatocytes with *Setdb1* deficiency displayed aberrant centromere clustering and bouquet formation, failure of homologous chromosome pairing and synapsis, and impaired meiotic silencing of unsynapsed chromatin, leading to meiotic arrest before pachytene and spermatocytes apoptosis [68, 69]. In addition, SETDB1 was also identified as a maternal transcriptional co-regulator of genes contributed to mitosis during early embryos in mice [70].

In *Drosophila*, H3K9me3 chromatin has been found to be critical for the maintenance of female germ cell fate [71]. SETDB1, its binding partner Windei/Wed and HP1a were found to be necessary for silencing testis gene transcription (e.g., *phf7*) by enhancing H3K9me3 at these genes in female germ cells [71]. In mice, SETDB1 has been reported to act as a bridge between the meiotic DNA damage response and sex chromosome silencing, and meiotic *Setdb1* deletion induces midpachytene apoptosis by perturbing meiotic sex chromosome remodeling and silencing during male meiosis [69]. Additionally, SETDB1 has been found to associate with the topological regulator Cohesin to regulate embryonic stem cell pluripotency and lineage development by affecting the topological structures of related genes [25, 72].

#### Embryonic stem cell

**Fig. 3:**
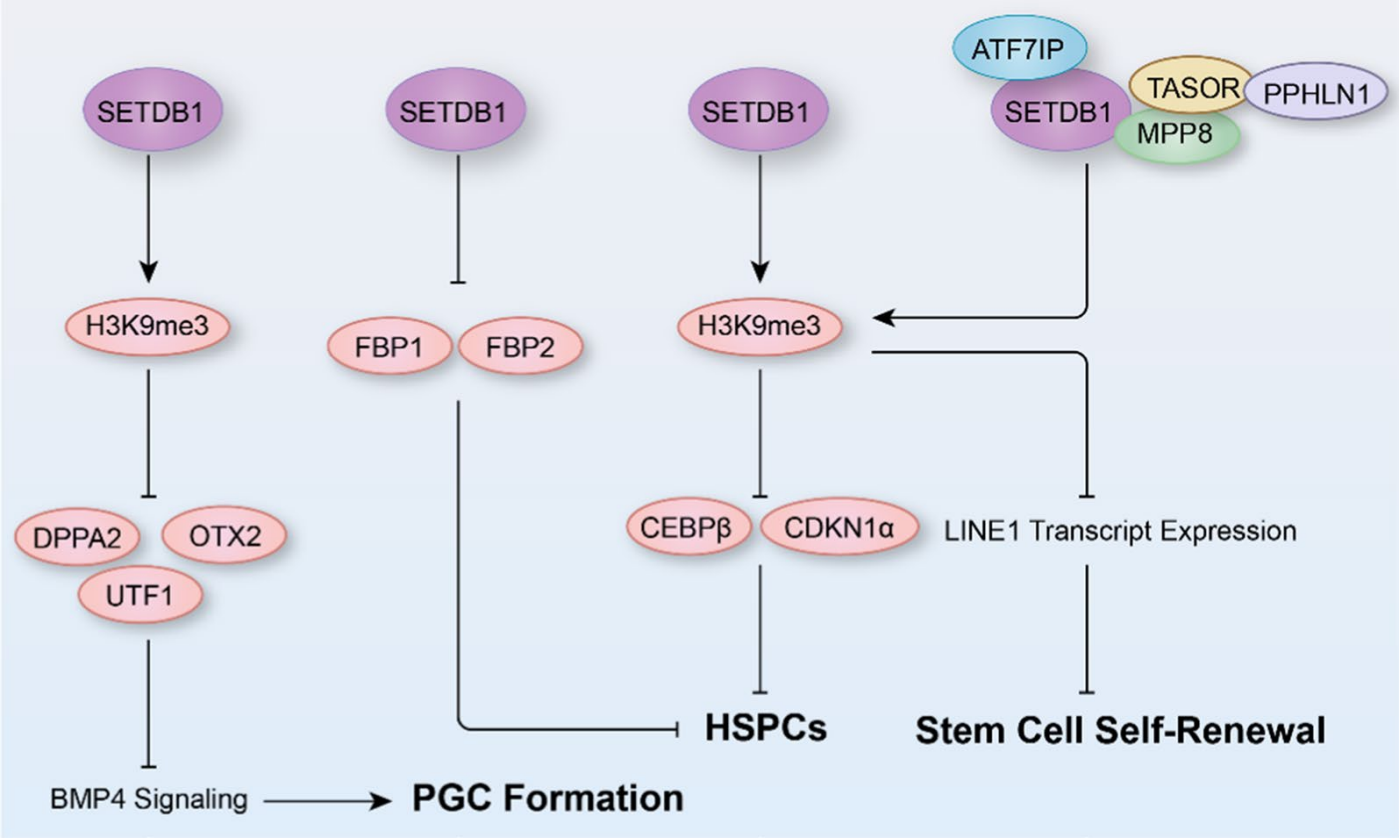
The mechanisms underlying the role of SETDB1 in cell fate determination and stem cell. SETDB1 trimethylates H3K9 (H3K9me3) to suppress the expression of DPPA2, OTX2, and UTF1 to regulate primordial germ cells (PGC) formation, and to inhibit CEBPβ and CDKN1α, FBP1 and FBP2 expression to affect the hematopoietic stem and progenitor cells (HSPCs), and to repress LINE1 transcript expression to facilitate stem cell self-renewal

Retrotransposons constitute nearly forty percent of the mouse genome [73], and are mostly transcriptionally silenced during early embryogenesis [74]. In mESCs and in early embryos, deletion of KAP1 (also known as TRIM28), a partner of SETDB1, led to a marked upregulation of a range of ERVs, particularly intracisternal A-type particles (IAP) elements, by downregulating H3K9me3 levels in these regions [23, 74]. Germline-specific conditional knockout of *Setdb1* produced a reduction in methylated long terminal repeats (LTRs) and LINE1 elements, as well as DNA methylation at H3K9me3-enriched retrotransposon families, which results in concomitant derepression of marked IAP, ETn, and ERVK10C elements, as well as ERV-proximal genes in *Setdb1* deficiency E13.5 primordial germ cells (PGCs) of mice [73]. Interestingly, *Setdb1* knockout mice showed a reduced number of male E13.5 PGCs, while postnatal hypogonadism in both sexes [73]. Furthermore, SETDB1 has been found to inhibit the expression of *Dppa2* (developmental pluripotency associated 2), *Otx2* (orthodenticle homeobox 2) and *Utf1* (undifferentiated embryonic cell transcription factor 1), while activating the BMP/SMAD pathway genes *Acvrl1* and *Smad* in developing PGCs, which indicates that SETDB1 is crucial for PGC fate determination of epiblast cells (Fig. 3) [75].

Although histone methylation and DNA methylation are essential for the repression of ERVs transcription, the genes upregulated after *Setdb1* deletion differ from those derepressed genes in mESCs with *Dnmt1*, *Dnmt3a*, and *Dnmt3b* deficient, with the exception of a small number of primarily germline-specific genes [22]. This paradoxical phenotype may be due to an ectopic interaction between SETDB1 and NP95/UHRF1. Under normal conditions, SETDB1 maintains silencing of ERVs, while in the absence of DNMT1, prolonged binding of NP95/UHRF1 to hemimethylated DNA transiently disrupts SETDB1-dependent deposition of H3K9me3 in these regions [76]. In naïve ESCs with SETDB1 deficiency, Tet methylcytosine dioxygenase 2 (TET2) activates IAP elements in a catalytic-dependent manner. Surprisingly, TET2 has no effect on DNA methylation levels at IAPs, but regulates these retrotransposons in a TET2-dependent loss of H4R3me2s manner [77]. In addition, the physically interaction between SETDB1 and methyltransferase-like 3 (METTL3), an RNA N6-methyladenosine (m^6^A) methyltransferase, is important for the integrity of IAP heterochromatin in mESCs [78–81].

#### Hematopoietic stem and progenitor cell

Additionally, *Setdb1* has been found to be critical for the maintenance of hematopoietic stem and progenitor cells (HSPCs) in mice, as demonstrated by the rapid depletion of hematopoietic stem, HSPCs, and leukemic stem cells after *Setdb1* deletion, which is caused by ectopic activation of nonhematopoietic genes (e.g., gluconeogenic enzyme genes fructose-1,6-bisphosphatase 1 (*Fbp1*) and *Fbp2*) (Fig. 3) [82]. Conditional ablation of *Setdb1* in pro-B cells (Mb1-Cre) has a significant impact on the pro-B cell compartment, and the B cell populations in the spleen and bone marrow that correspond to later developmental stages are virtually eliminated in mice [83]. Further evidence suggests that these effects of *Setdb1* deficiency on B cells may be associated with derepression of endogenous murine leukemia virus (MLV) copies and subsequent activation of the unfolded protein response pathway and apoptosis [84]. In addition, SETDB1 limits the priming of T helper 1 (Th1) cells and maintains the integrity of Th2 cells by repressing a repertoire of ERVs in a H3K9me3-dependent manner [85].

Similarly, in zebrafishes with *Setdb1* or *Atf7ip* deficiency, excessive myeloid differentiation with impaired HSPC expansion is observed, leading to a decrease in T cell and erythroid lineage [86]. Mechanistically, *Setdb1* and *Atf7ip* interaction facilitates H3K9me3 deposition in *cebpβ* and *cdkn1a* to inhibit their expression (Fig. 3) [86]. Concomitantly, deletion of *Atf7ip* or *Setdb1* derepresses retrotransposons, thereby inhibiting human leukemia cell growth and inducing myeloid differentiation and inflammation by activating the viral sensor Mda5/Rig-I like receptor signaling [86].

#### Other stem cell

Fei et al*.* demonstrated that SETDB1 works in coordination with Polycomb repressive complex 2 (PRC2) to suppress neural differentiation independently of H3K9me3 [87]. Moreover, ERVs are heavily DNA methylated in both ESCs and differentiated somatic cells, but distinctive sets of ERVs are reactivated in different types of *Setdb1*-deficient somatic cells in an H3K9me3-dependent or -independent manner [21].

In conclusion, SETDB1 plays an important role in cell fate determination, stem cell differentiation and function, but the regulatory mechanisms are heterogeneous and cell-specific. For example, transcriptomic results showed that *Setdb1* deletion significantly induced the expression of ERV families such as the murine leukemia virus (MLV), mouse mammary tumor virus (MMTV) and VL30 in pro-B cells, whereas these ERVs remained silent or expressed at low levels in SETDB1-deficient ESCs and PGCs, which expressed ERV families such as IAPE-z, GLn and ETn/MusD [83]. Thus, further studies are needed to further elucidate the specific mechanisms by which SETDB1 regulates cell fate determination and stem cell differentiation, and how this cell-specific mechanism is achieved, e.g., is it dependent on H3K9me3? and which regulators are involved in determining this cell-specific mechanism.

### SETDB1 in tumors

**Fig. 4:**
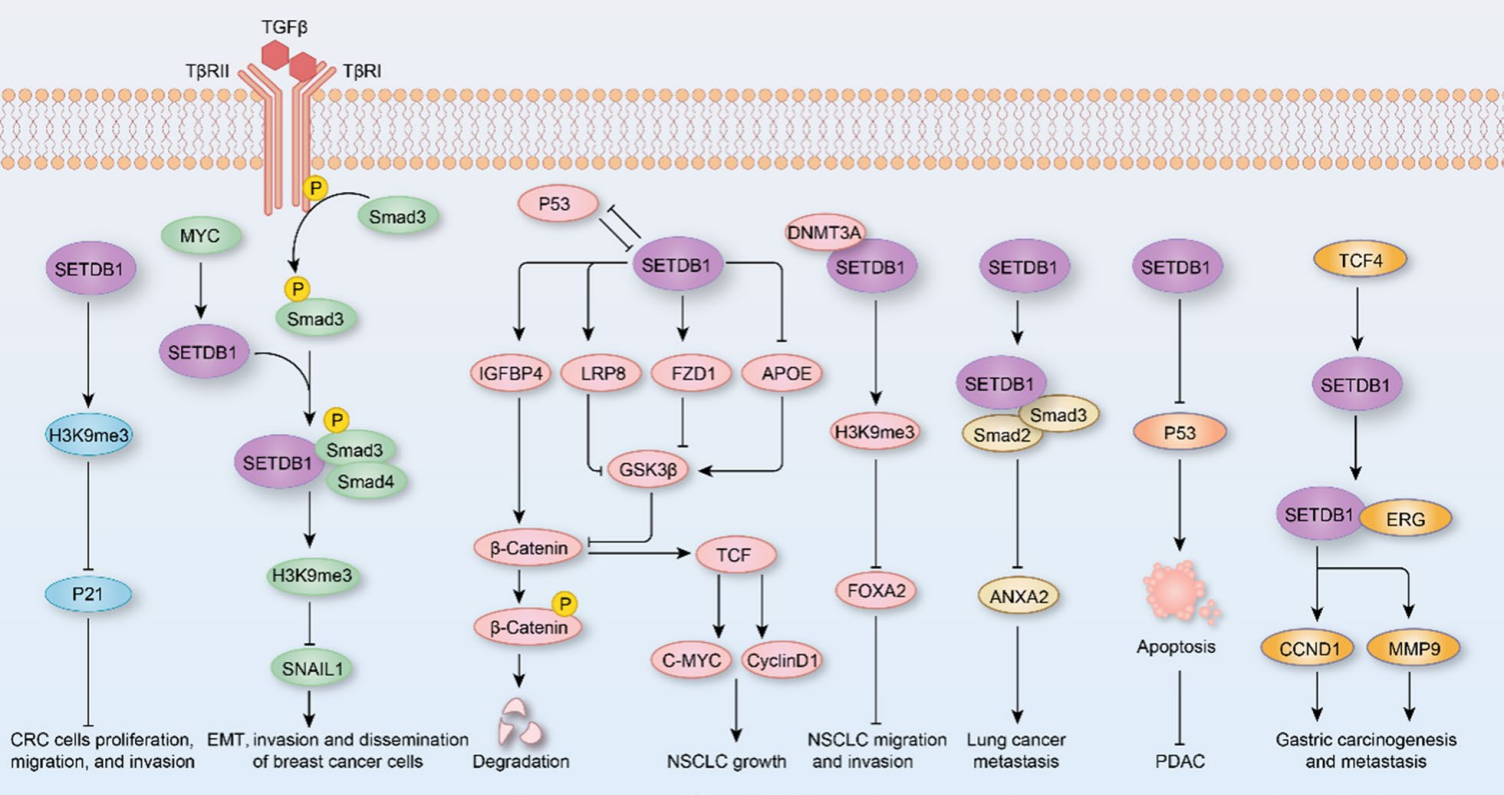
The mechanisms mediating the function of SETDB1 in tumors. SETDB1 directly inhibits or cooperates with other modulators (e.g., Smad3 and ERG) to regulate the genes-related to tumorigenesis and tumor progression. *CRC* colorectal cancer, *EMT* epithelial–mesenchymal transition, *NSCLC* non-small cell lung cancer, *PDAC* pancreatic ductal adenocarcinomas

According to Global Cancer Statistics, there were an estimated 19.3 million new cancer cases and 10 million cancer-related deaths worldwide in 2020, with lung cancer being the most commonly diagnosed cancer in both sexes [88]. SETDB1 has been found to be amplified in lung cancer cell lines and primary tumors, resulting in increased mRNA and protein levels, which contributes to tumor growth and invasion [89]. SETDB1 promotes the expression of IGFBP4 (insulin like growth factor binding protein 4), LRP8 (LDL receptor related protein 8), and FZD1 (frizzled class receptor 1), but inhibits APOE (Apolipoprotein E) expression, thereby activating WNT-β-catenin pathway and suppressing P53 expression to enhance NSCLC growth in vitro and in vivo [90]. Recently, Zakharova et al*.* demonstrated that SETDB1 plays an essential role in epigenome, 3D genome organization, and chromatin architecture in the determination of lung adenocarcinoma programs [91]. Due to its high expression in lung cancer, SETDB1 can be used as a diagnostic biomarker for NSCLC with an area under the curve of 0.7741 [92]. SETDB1 also reinforces invadopodia formation and extracellular matrix degradation by suppressing forkhead box A2 (FOXA2) expression, which facilitates migration and invasion capabilities of NSCLC cells [93]. However, Wu et al*.* demonstrated that in highly metastatic lung cancer cells, SETDB1 is downregulated and interacts with SMAD2/3 to repress metastasis of lung cancer through ANXA2 (Annexin A2) [94]. Similarly, during breast cancer, TGF-β activates SMAD3, which recruits SETDB1 to methylate H3K9, while repressing H3K9 acetylation to inhibit the transcription of* Snai1* (snail family transcriptional repressor 1) gene [95]. SNAIL1 is the “master” transcription factor that regulates epithelial–mesenchymal transition (EMT), cancer stem cell properties, cancer dissemination, and patient survival [96]. Thus, SETDB1 impairs TGF-β-induced EMT by interacting with SMAD3 and then downregulating SNAIL1 (Fig. 4). In squamous cell carcinoma, enhancer of zeste homolog 2 (EZH2) inhibits RUNX3 (RUNX Family Transcription Factor 3) expression to activate both SETDB1 and ΔNp63α, which drives an aggressive cancer stem cell phenotype [97]. In contrast, Xiao et al*.* demonstrated that SETDB1 facilitates the translation of c-MYC and Cyclin D1 mRNAs to promote cell cycle progression of breast cancer cells. Moreover, c-MYC directly binds to the promoter regions of *Bmi1* and* Setdb1* to enhance their expression, suggesting a positive feedback loop between SETDB1 and c-MYC [40]. Overall, these studies indicate that SETDB1 may act as an oncogenic driver, but is a tumor metastasis suppressor in lung and breast cancers (Fig. 4).

In hepatitis B-associated human HCC, SETDB1 is upregulated and has been linked to HCC disease progression and poorer prognosis [98]. Sp1, a positive regulator of SETDB1, is hyperactivated, while miR-29, a negative regulator of SETDB1 was downregulated in HCC, and they jointly contribute to the high expression of STEDB1 [98]. In addition to miR-29, miR-621 has been shown to reduce the expression of SETDB1 by directly targeting its 3′ UTR and enhancing the radiosensitivity of HCC cells, which is mediated by the P53 signaling pathway [42]. Furthermore, *Setdb1* deficiency has been found to increase apoptosis to prevent formation of pancreatic ductal adenocarcinomas (PDACs) by binding to p53 promoter regions and directly regulating its expression in the context of heterozygous p53 deletion (Fig. 4) [99].

SETDB1 has been found to be highly amplified in tumors of melanoma patients and melanoma cell lines, with overexpression contributing to a more aggressive phenotype in vivo and in vitro studies by upregulating thrombospondin 1 (THBS1) expression in a H3K4me1-dependent manner [100]. Ceol et al*.* demonstrated that SETDB1 accelerates formation of melanoma in a zebrafish model by trimethylating H3K9, which binds to the HOXA genes to enhance its expression [101]. Aberrant overexpression of SETDB1 is also detected in colorectal cancer (CRC), promoting CRC cell proliferation, migration, and invasion [102]. However, SETDB1 deficiency arrests CRC cells in G1 phase to inhibit cell proliferation and CRC tumorigenesis by decreasing H3K9me3 enrichment at the promoter of *p21* and then suppressing p21 expression (Fig. 4) [102]. Similarly, *Helicobacter pylori* (*H. pylori*) infection induces SETDB1 expression in a TCF4-dependent manner, which contributes to gastric cancer formation [39]. Increased SETDB1 directly interacts with ERG (ETS transcription factor) to facilitate the expression of CCND1 and MMP9 to promote cell proliferation and metastasis (Fig. 4) [39]. Co-administration of CDK4/6 inhibitor palbociclib obviously enhanced the therapeutic efficacy of SETDB1 depletion on tumor growth by protecting TRIM28-mediated p-RB from proteasomal degradation both in vitro and in vivo [103]. On the contrary, SETDB1 inhibits the expression of genes associated with acute myeloid leukemia (AML), such as *Dock1*, *Hoxa9*, and *Six1*, to delay MLL-AF9-mediated disease progression by promoting the differentiation of leukemia cells [104].

It is reported that the protein levels of H3K9me3 and SETDB1 were increased in patients with pediatric high-grade gliomas [105]. SETDB1 silent attenuated cell viability, proliferation and migration, while increased apoptosis in two patient-derived high-grade gliomas cell lines [105]. The same conclusion was reached in another study using glioma cell lines (GOS-3, 1321N1, T98G, and U87MG) [106]. In this study, the authors also showed that another H3K9me3 methyltransferase, SUV39H1, also has the ability to regulate glial cell proliferation, migration, and colony-forming capacity [106]. Similarly, inhibition of SUV39H1 by Chaetocin significantly inhibited proliferation, clonogenic potential, and migration ability of T98G cells [107]. Furthermore, increased nuclear SUV39H1 expression was correlated with adverse prognosis of glioblastomas patients [107]. Thus, these studies indicated that H3K9me3, as well as its methyltransferases SETDB1 and SUV39H1, play important regulatory roles in glioma development and progression and may represent novel targets for targeted therapy of glioma.

Immunotherapies have shown considerable efficacy in treating several types of cancer. Amplification of SETDB1 in human tumors has been reported to contribute to immune exclusion and resistance to immune checkpoint blockade [108]. Screening chromatin regulators by CRISPR-Cas9, Griffin et al*.* identified that SETDB1 and other members of the HUSH and KAP1 complexes act as mediators of immune escape in a mouse tumor model treated with immune checkpoint blockade [109]. They further found that SETDB1 represses broad domains that are enriched in immune clusters and transposable elements associated with segmental duplication events, thereby suppressing transposable element-encoded retroviral antigens, latent transposable element-derived regulatory elements, immunostimulatory genes, and triggering transposable element-specific cytotoxic T cell responses in vivo [109]. Monoclonal antibodies (mAbs) targeting the programmed cell death protein 1/programmed cell death ligand 1 (PD-1/PD-L1) axis have shown striking clinical benefit in several types of cancer [110]. SETDB1-TRIM28 inhibition combined with elevated PD-L1 facilitated the formation of micronuclei in the cytoplasm, thereby activating the cGAS (cyclic GMP-AMP synthase)-STING (stimulator of interferon genes) innate immune response pathway and increasing infiltration of CD8^+^ T cells [108]. Furthermore, SETDB1 deficiency has been found to improve the antitumor effects of anti-PD-L1 [108]. Lysine Demethylase 5B (KDM5B) recruits SETDB1 to inhibit endogenous retroelements in a demethylase-independent manner, thereby inhibiting the cGAS-STING pathway and type-I interferon response, which contributes to tumor growth and immune memory inhibition [97]. Similarly, in acute myeloid leukemia, SETDB1 has been found to function as a novel negative innate immune regulator by suppressing type I interferon response, thereby contributing to immune evasion and oncogenic cellular state [111]. In NSCLC and melanoma patients, SETDB1 expression level is shown to be negatively correlated with radiotherapy efficacy. Moreover, SETDB1 inhibition markedly improves radiotherapy efficacy by facilitating the expression of basal and radiation-induced ERVs, enhancing MDA5/MAVS signaling, and upregulating type I interferons expression [112]. These studies suggest that SETDB1 is a negative regulator of tumor-intrinsic immunogenicity and thus a potential target for immunotherapy.

### SETDB1 in other biological processes and diseases

It has been reported that approximately 10–15% of tumor cells lengthen telomeres by the alternative lengthening of telomeres (ALT) mechanism, in which TRIM28 protects the telomeric histone methyltransferase SETDB1 from degradation, thereby maintaining the H3K9me3 heterochromatin state of the telomeric DNA, which prevents telomere shortening and reduces telomeric sister chromatid exchange in cells [113]. Consistently, SETDB1 deficiency disrupts telomeric heterochromatin and abrogates ALT [114]. Telomere length is strongly associated with aging, which is the greatest risk factor for cancer. Hallmarks of aging include genomic instability, telomere attrition, mitochondrial dysfunction, cellular senescence, epigenetic alterations, loss of proteostasis, deregulated nutrient sensing, stem cell exhaustion, and altered intercellular communication [115]. The expression of SETDB1 is decreased significantly with age, and SETDB1 affects aging by regulating mitochondrial function [116]. Aging also causes a reduction in the function of spermatogonial stem cell and an increase in the risk of paternal age-related genetic diseases. SETDB1 deficiency impairs cell proliferation and cell–cell adhesion in spermatogonial stem cells by depositing H3K9me3 to inhibit MMP3/10 expression [117].

Chronic inflammation in the gastrointestinal tract is a key feature of inflammatory bowel disease (IBD), and patients with IBD are at increased risk of developing CRC [118, 119]. The expression level of SETDB1 is decreased and rare missense variants of SETDB1 are over-represented in patients with IBD [120, 121]. Moreover, *Setdb1* deficiency in mouse intestinal epithelial cells is associated with barrier disruption, defective intestinal epithelial differentiation, inflammation and mortality, indicating that SETDB1 is essential for intestinal epithelial homeostasis [120]. Furthermore, mice with downregulated SETDB1 expression in intestinal stem cells develop spontaneous terminal ileitis and colitis by triggering Z-DNA-binding protein 1 (ZBP1)-dependent necroptosis via de-silencing endogenous retroviruses [121]. Thus, targeting SETDB1 or necroptosis of intestinal stem cells may be potential novel strategies for the treatment of severe IBD in humans. Interestingly, conditional knockout of Setdb1 (Setdb1-NS-cKO) in mouse neural progenitor cells showed that SETDB1 represses 5-hydroxytryptamine receptor 3A (*Htr3a*) transcription through endogenous retroviral sequence RMER21B-mediated distal chromatin interactions in the embryonic ganglionic eminence, thereby modulating mood behaviors and cortical Htr3a-positive interneurons development [122]. Moreover, SETDB1 was also reported to be involved in neural progenitor cells switch differentiation, and forebrain-specific *Setdb1* knockout mice showed early neurogenesis impairment, severe brain defects and early lethality [123]. Additionally, SETDB1 and ATF7IP were found to cooperatively promote osteoblast proliferation by catalyzing H3K9me3 in Macrod2 (mono-ADP ribosylhydrolase 2) promoter region to suppress its expression under mechanical unloading [124]. Therefore, these studies indicated that SETDB1 is a versatile epigenetic regulator.

## The functions of SETDB1 by methylating non-histone proteins

**Fig. 5:**
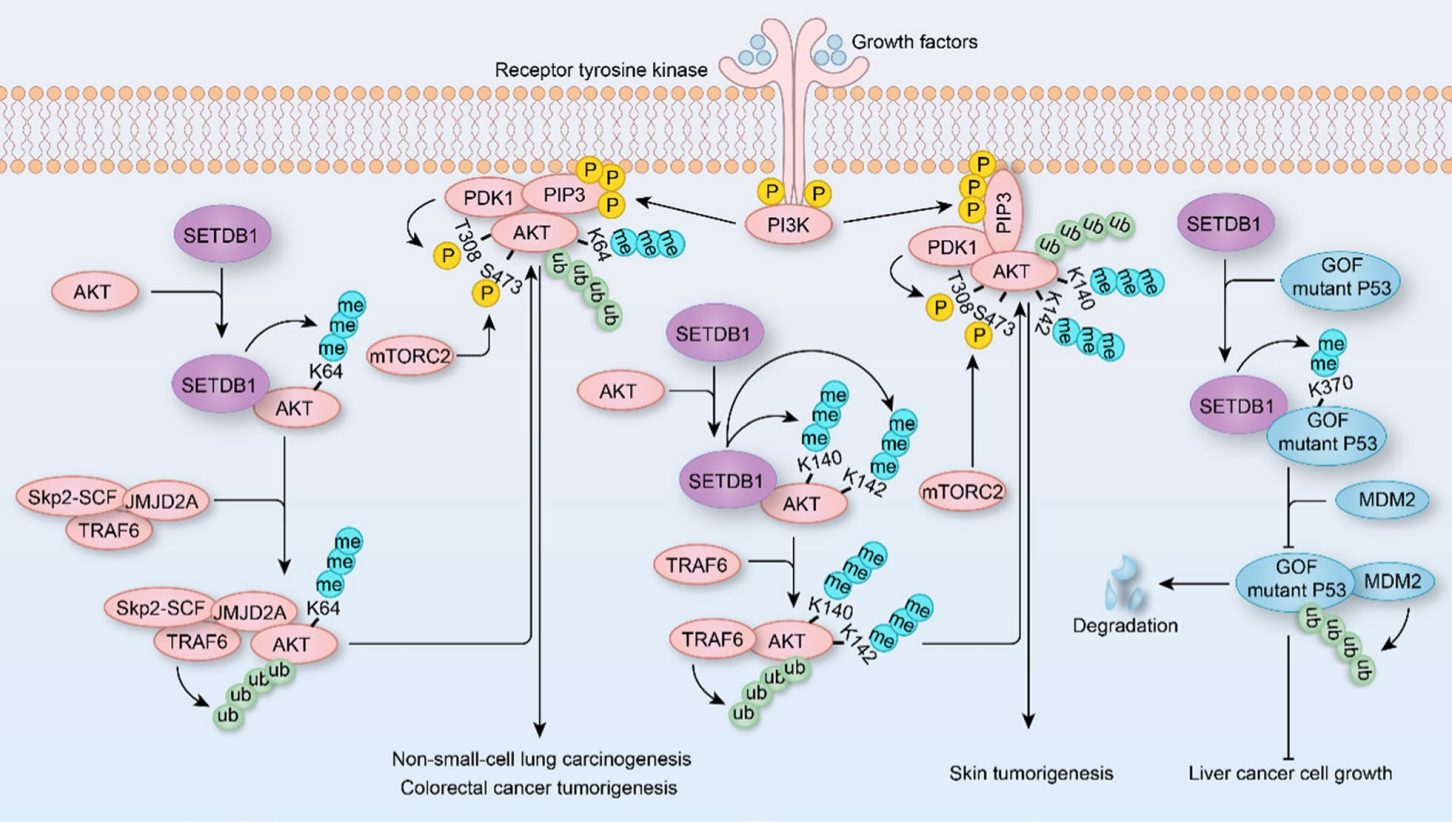
The role of SETDB1 in tumors by methylating nonhistones. SETDB1 trimethylates AKT at K64, K140 or K142 to increase the phosphorylation and activity of AKT, which accelerates non-small cell lung cancer and colorectal cancer or skin tumorigenesis, respectively. In liver cancer, SETDB1 dimethylates gain-of-function (GOF) mutant P53 at K370 to prevent its degradation by ubiquitination, and then promotes liver cancer cell growth

In addition to histone methylation, accumulating evidence has demonstrated that non-histone proteins can also be methylated by histone methyltransferases, for example, histone methyltransferase SMYD2 mono-methylates K370 site of P53 in tumor cells [3, 125, 126]. SETDB1 is known to be not only a methyltransferase that tri-methylates H3K9 but also methylates non-histone proteins (e.g., P53 and AKT) to participate in tumorigenesis (Fig. 5) [17–19, 127]. Moderate copy number gain of SETDB1 leads to its overexpression in HCC, which catalyzes the K370 site of P53 di-methylation by interacting with P53 to increase recognition and degradation of P53 by MDM2 [19]. In addition, SETDB1 binds to Trp53 promoter to inhibit its expression, decreasing apoptosis and increasing growth of human PDACs [99]. These studies indicate that SETDB1 kills two birds with one stone, that is, SETDB1 not only suppresses P53 expression at the transcriptional level, but also methylates P53 to promote its degradation, and then facilitating tumor development.

In addition to P53, AKT is also a substrate that is methylated by SETDB1 and involved in tumorigenesis (Fig. 5) [17, 18, 127]. SETDB1 tri-methylates the K140 and K142 sites of AKT to promote its phosphorylation on T308 and S473 sites and activation, which is antagonized by lysine demethylase 4B (KDM4B). Moreover, non-methylated mutant *Akt1* knock-in mice not only have reduced body size and weight, but also less susceptible to carcinogen-induced skin tumorigenesis [18]. In NSCLC, SETDB1 triggers di- and tri-methylation of the AKT K64 site followed by initiation of K63-linked AKT ubiquitination by recruiting Jumonji Domain-Containing Protein 2A (JMJD2A) and E3 ligases (e.g., TRAF6 and Skp2-SCF) to the AKT complex, which in turn leads to cell membrane recruitment, T308 phosphorylation and subsequently activation of AKT. AKT hyperactivation promotes NSCLC progression and predicts poor outcome in NSCLC patients [17]. Similarly, in colorectal cancer, SETDB1 overexpression facilitates cell proliferation by activating AKT, and inhibition of SETDB1 augments the sensitivity of cetuximab in colorectal cancer [127]. Therefore, targeting SETDB1-mediated AKT methylation is a promising strategy for the treatment of cancers (such as skin tumor, NSCLC, and colorectal cancer).

## Conclusions and perspectives

The recent surge of studies on SETDB1 has highlighted its vital role in regulating multiple diseases, embryonic development, and stem cells by methylating H3K9 or non-histone proteins. SETDB1 plays an indispensable role in cell fate determination by regulating ERVs silencing in H3K9 methylation-dependent or -independent manner. However, further research is needed to understand how SETDB1 works with different partners to regulate cell fate under different pathophysiological conditions. Interestingly, SETDB1 has been found to be upregulated in most cancers, and its overexpression has been significantly associated with cancer aggressiveness and poorer prognosis. However, in some cancers, SETDB1 functions as an oncogenic driver, while in others it is a tumor metastasis suppressor. The relevant molecular mechanisms deserve further exploration. Although some studies have revealed the role of SETDB1 in diseases such as IBD, the effects and regulatory mechanisms of SETDB1 on diseases need to be further elucidated. In addition to methylating H3K9, some recent studies have found that SETDB1 can methylate non-histone proteins (e.g., P53 and AKT), which further expands the downstream mechanisms of SETDB1 and deepens our understanding of SETDB1. There is an urgent need to clarify the function of SETDB1 under distinct physiological or pathological conditions and to ascertain how the specificity of SETDB1 binding to a specific substrate (histones or non-histone proteins) is determined. Most importantly, SETDB1 inhibition has been found to significantly enhance the efficacy of radiotherapy and immunotherapy in cancers, suggesting that the development of SETDB1 inhibitors/drugs with high specificity, low toxicity, and high efficiency could provide new options for tumor treatment by targeting SETDB1. Additionally, since SETDB1 is an important regulator that controls the development of many diseases, therapeutic RNAs targeting SETDB1, including small interfering RNAs (siRNAs), antisense oligonucleotides (ASOs), or large RNAs such as mRNAs, long non-coding RNAs (lncRNAs), and cyclic RNAs, represent an alternative strategy for developing treatments for diseases.

## Data Availability

All the materials will be provided from the corresponding author on reasonable request.

## Abbreviations

SETDB1: SET domain bifurcated histone lysine methyltransferase 1
ERVs: Endogenous retroviruses
mESCs: Embryonic stem cells
DNMTs: DNA methyltransferases
IBD: Inflammatory bowel disease
AdoMet: *S*-Adenosyl-l-methionine
ESET: Erg-associated SET domain
KMT1E: Lysine *N*-methyltransferase 1E
HP1a: Heterochromatin protein 1a
TUDs: Tudor domains
MBD: Methyl-CpG binding domain
Sp1: Specificity protein 1
MAPK: Mitogen-activated protein kinase
TCF4: Transcription factor 4
GC: Gastric cancer
MMP9: Matrix metalloproteinase 9
CCND1: Cyclin D1
HCC: Hepatocellular carcinoma
NSCLC: Non-small cell lung cancer
ATF7IP: Activating transcription factor 7-interacting protein
CAF1: Chromatin assembly factor 1
KAP-1: KRAB-ZFP-associated protein 1
IAP: Intracisternal A-type particles
LTRs: Long terminal repeats
PGCs: Primordial germ cells
Dppa2: Developmental pluripotency associated 2
Otx2: Orthodenticle homeobox 2
Utf1: Undifferentiated embryonic cell transcription factor 1
MPP8: M-phase phosphoprotein 8
TET2: Tet methylcytosine dioxygenase 2
HSPCs: Hematopoietic stem and progenitor cells
METTL3: Methyltransferase-like 3
PRC2: Polycomb repressive complex 2
IGFBP4: Insulin like growth factor binding protein 4
LRP8: LDL receptor related protein 8
FZD1: Frizzled class receptor 1
APOE: Apolipoprotein E
FOXA2: Forkhead box A2
ANXA2: Annexin A2
EMT: Epithelial–mesenchymal transition
Snai1: Snail family transcriptional repressor 1
EZH2: Enhancer of zeste homolog 2
RUNX3: RUNX Family Transcription Factor 3
PDACs: Pancreatic ductal adenocarcinomas
THBS1: Thrombospondin 1
CRC: Colorectal cancer
AML: Acute myeloid leukemia
PD-1: Programmed cell death protein 1
PD-L1: Programmed cell death ligand 1
cGAS: Cyclic GMP-AMP synthase
STING: Stimulator of interferon genes
KDM5B: Lysine demethylase 5B
ALT: Alternative lengthening of telomeres
ZBP1: Z-DNA-binding protein 1
Htr3a: 5-Hydroxytryptamine receptor 3A
Macrod2: Mono-ADP Ribosylhydrolase 2
KDM4B: Lysine demethylase 4B
JMJD2A: Jumonji Domain-Containing Protein 2A
